# Dual-function strategy: simultaneous biopolymer production and lead biosorption by *Bacillus paramycoides* in single-batch fermentation process

**DOI:** 10.1007/s10532-025-10160-2

**Published:** 2025-07-22

**Authors:** Raghavendra Paduvari, Roopashri Arekal, Divyashree Mysore Somashekara

**Affiliations:** 1 Department of Biotechnology, Manipal Institute of Technology, Manipal Academy of Higher Education, Manipal, 576104 Karnataka India; 2 Department of Microbiology, Biotechnology and Food Technology, Bangalore University, Bengaluru, 560056 Karnataka India

**Keywords:** Biosorption, Bioadsorption, Bioremediation, Lead, *Bacillus Paramycoides*, Polyhydroxyalkanoate

## Abstract

The conventional physicochemical processes of lead removal from contaminated wastewater are cost-intensive and generate toxic secondary wastes. Therefore, employing bacteria capable of lead biosorption will serve as a green, sustainable, safe and inexpensive process for lead removal, and its integration with polyhydroxyalkanoate (PHA) production will generate revenue. To integrate these two processes, *Bacillus paramycoides* isolated from the lake water were analyzed for lead biosorption and PHA production in mineral salt media containing lead nitrate at different incubation times. The bacterium showed a high lead biosorption of about 423 mg/g of biomass at 72 h of incubation, which was earlier not reported. *Bacillus paramycoides* produced polyhydroxybutyrate by utilizing sucrose in the media. The lead ions enhanced the PHA production up to 35.7% CDW in bacterial cells by inducing oxidative stress, and the ability of PHA polymer to adsorb lead ions prevented further oxidative damage to the cells. This study is the first report on using an integrated process approach for lead biosorption and PHA production in bacteria.

**Supplementary Information:**

The online version contains supplementary material available at 10.1007/s10532-025-10160-2.

## Introduction

Despite their toxicity, the excess use of heavy metals for industrial and commercial purposes has devastated the environment. Industrial effluents, domestic wastes and leachates carry heavy metals in ionic or non-ionic form to the soil and water bodies, contaminating the ecosystems. Since heavy metals pose a threat in their ionic form, there is no possibility of further degradation, leading to toxication and lethal effects after ingestion (Alissa and Ferns 2011). Heavy metals enter living organisms mainly through water and food ingestion. It persists in various living organisms without any change and traverses multiple food chain trophic levels, causing biomagnification. Lead is among the most toxic heavy metal pollutants that have emerged due to anthropogenic causes (Priyadarshanee and Das 2021). The widespread use of lead in batteries, refineries, pigments, paints, metal plating, preparation of metal alloys and electric power plants has increased the risk of pollution due to lead. The untreated domestic and industrial effluents enter various water bodies, leading to lead pollution(Sevak et al. 2021). Certain inefficient and cost-intensive purification strategies, such as filtration and precipitation adapted for lead removal from drinking water, result in a high residual lead concentration, causing lead intoxication and lethal effects(Ansari et al. 2011). Lead is known to cause oxidative stress in various organisms by generating free radicals, and it is classified as a mutagenic and teratogenic agent(Kumar et al. 2022). It is known to cause encephalopathy, renal failure, cancer, hepatitis, anaemia, gastrointestinal diseases, and cardiovascular and neurodegenerative disorders in humans. It also disrupts plant physiology and affects chlorophyll, seed germination, transpiration and root development, causing stunted plant growth(Sevak et al. 2021). Therefore, an efficient lead removal strategy is necessary to purify drinking water and treat wastewater for commercial use. The bacterial consortia containing *Pseudomonas* sp., *Bacillus* and *Rhodococcus* sp. have been currently used in treatment of municipal sewage water, mainly due to its capability to degrade and growth in wide range of toxic aliphatic and aromatic hydrocarbons(Muter 2023). Hence, bacterial-based waste water treatment serves as a cost-effective and green technique for water purification.

Lead removal from contaminated water involves various physicochemical methods such as electrocoagulation, electrolysis, photocatalysis, ultrafiltration, precipitation, osmosis and adsorptive processes using activated charcoal that are cost-intensive and generate harmful secondary wastes(Ansari et al. 2011). Biosorption is a strategy that employs living or dead biomass and organism-derived materials as a biosorbent for removing lead from contaminated water. Biosorption is a collective term that involves various processes, such as chelation, ion exchange, adsorption, and microprecipitation(Priyadarshanee and Das 2021; Ansari et al. 2011). Bacterial lead biosorption is a cost-effective lead removal strategy for contaminated water treatment mainly due to the ease of bacterial cultivation using defined mineral salts, inexpensive carbon substrates, the ability to achieve high biomass and reduced generation of non-toxic secondary wastes. The technique minimizes the harmful release of chemical sludge and helps recover lead from biomass(Hansda et al. 2016). The bacterial biosorption of heavy metals has been extensively studied previously in the *Bacillus* sp., *Pseudomonas* sp., and *Azotobacter* sp. *Bacillus subtilis* BM2, *Pseudomonas aeruginosa* CPSB1 and *Azotobacter chroococcum* CAZ3 dried and powdered biomass was used in biosorption of copper (Cu), cadmium (Cd), zinc (Zn), nickel (Ni) and chromium (Cr) from contaminated water(Rizvi et al. 2020). *Pseudomonas alcaliphila* NEWG-2 immobilized bacterial biomass has been demonstrated to adsorb Cr^+6^ ions from water(El-Naggar et al. 2020). However, the inefficiency of bacterial strains in absorbing and retaining lead during growth in media limits the application of bacterial lead biosorption(Ansari et al. 2011). Hence, the present study involves developing *Bacillus paramycoides*, isolated from Manipal Lake water, as an efficient bacterial biosorbent for removing lead from contaminated water.

Integrative approaches are widely used to obtain various value-added products during bacterial cultivation due to their cost-effectiveness and increased revenue generation, which can be achieved by producing multiple products in a single process. Various integrative approaches have synthesized polyhydroxyalkanoates (PHA) and other sustainable products(Katagi et al. 2024). PHA are biodegradable bioplastics produced by a few bacteria intracellularly during growth in excess carbon source and nutrient-limiting conditions. The PHA are polymers of (*R*)-3-hydroxyalkanoic acids held by ester linkages(Kumar et al. 2020). These bioplastics have wide industrial, biomedical, and agricultural applications. It can be used in packaging material, single-use plastics, drug delivery systems, wound dressing material, body implants, and preparation of surface-coating materials and polymer composites(Muneer et al. 2020; Dwivedi et al. 2020). The production of PHA and biosorption of lead in bacteria during cultivation has not been explored so far. Hence, the present study explores the biosorption of lead and simultaneous production of PHA as an integrative process approach in *Bacillus paramycoides* cultured in mineral salt media containing lead.

## Materials and methods

### Collection of water sample, chemical constituent analysis and bacterial isolation

The water sample was collected from Manipal Lake (Location coordinates: 13°20′25.3"N 74°47′08.8"E) using a sterile 5 L container and tightly sealed. The chemical parameters such as chemical oxygen demand (COD), biological oxygen demand (BOD), dissolved oxygen, salinity, alkalinity, acidity, nitrates and nitrites were analyzed using standard methods (ESM1). The water sample was serially diluted from 10^–1^ to 10^–6^ under sterile conditions, and 100 µL of each was plated on a nutrient agar plate. The plates were incubated at 30 °C for 24 h. The bacterial colonies were analyzed for PHA production.

### Identification of bacteria

The bacterium capable of producing PHA was sent to the National Centre for Microbial Resource (NCMR), National Centre for Cell Science, Pune, Maharashtra, India, for identification using 16 s rRNA gene sequencing. The sequence homology of the 16 s rRNA gene sequence was analyzed using the BLAST analysis on the NCBI website, and the obtained results were used to construct a distance tree using the neighbour-joining method.

### Detection of PHA production in bacteria

The PHA production in bacterium was detected using Nile red staining of the viable bacterial colonies. The bacteria colonies were grown in PHA production agar media with a composition of Na_2_HPO_4_ (2.2 g/L), KH_2_PO_4_ (1.5 g/L), (NH_4_)_2_SO_3_ (1.5 g/L), Mg_2_SO_4_ 0.7H_2_O (0.2 g/L), sucrose (20 g/L) and agar (20 g/L) at 30 °C for 72 h. The bacterial colonies were flooded with 0.5 µg/mL Nile red dye solution in dimethylsulfoxide and illuminated under UV light (Spiekermann et al. 1999).

### PHA production

#### Preparation of media

The MSM was prepared by dissolving Na_2_HPO_4_ (2.2 g/L), KH_2_PO_4_ (1.5 g/L), (NH_4_)_2_SO_3_ (1.5 g/L), Mg_2_SO_4_·0.7H_2_O (0.2 g/L) and sucrose (20 g/L) in distilled water (Divyashree and Shamala 2010). For the media containing lead, 1 g/L of lead nitrate was added to the MSM. The pH was adjusted to 6.5 using 1 N NaOH solution and sterilized using an autoclave.

#### Preparation of inoculum and bacterial growth

A loop full of bacterial colonies was transferred to 10 mL nutrient broth taken in a 50 mL Erlenmeyer flask under aseptic conditions. The media was incubated at 30 °C, 170 rpm for 18 h. The bacterial inoculum was transferred to 100 mL of MSM taken in a 500 mL Erlenmeyer flask under aseptic conditions and incubated in a shaker incubator at 30 °C, 170 rpm.

### Estimation of bacterial biomass

The bacterial cultures in MSM were transferred to 50 mL centrifuge tubes at regular intervals and centrifuged at 8000 × g for 8 min to get the cell pellets. The pellets were dried in a hot air oven and the biomass was estimated gravimetrically.

### Estimation of PHA

The biomass was suspended in 4% sodium hypochlorite (1 mL/20 mg biomass) and incubated at 37 °C, 180 rpm for 1 h. The solution was diluted using distilled water and centrifuged at 8000 × g for 8 min at 4 °C to obtain the PHA pellets. The pellets were washed with distilled water and acetone to remove impurities. The PHA was estimated gravimetrically (Geethu et al. 2019).

### Gas chromatographic analysis of PHA

The PHA polymer of about 3 mg was dissolved in 1 mL of chloroform in an ampoule. To this solution, 750 µL of methanol and 250 µL of concentrated H_2_SO_4_ were added, and the ampoule was sealed. The methyl esters of the PHA monomers were synthesized after incubating the reaction mixture at 100 °C for 3 h in a glycerol bath. The seal was broken, and 500 µL of distilled water was added to the reaction mixture to get organic and aqueous layers. The organic layer of about 2 µL was injected into the PerkinElmer Clarus 590 gas chromatography equipped with a 30 m Elite-5 capillary column with an internal diameter of 0.25 mm and a flame ionization detector. Nitrogen was used as a carrier gas at a flow rate of 1 mL/min, and the temperature of the injector and detector were maintained at 200 °C and 220 °C, respectively. The oven temperature was initially kept at 55 °C for 7 min and increased to 100 °C at 4 °C/min. The oven was held at 100 °C for 1 min and increased to 200 °C at 10 °C/min. The oven was held at 200 °C for 10 min. The data was analyzed by comparing it with the standard polyhydroxybutyrate-co-valerate (PHBV)(Anil Kumar et al. 2007).

### Quantification of lead

#### Estimation of lead in culture supernatant

The bacterial cultures in MSM were harvested at regular intervals and centrifuged at 8000 × g for 8 min to get the culture supernatant. The supernatant was filtered through a 0.2 µm membrane using a sterile syringe, and the lead was estimated using atomic absorption spectroscopy (AAS)(Kushwaha et al. 2017).

#### Estimation of lead inside cells

The bacterial biomass obtained at regular intervals was digested in 4% sodium hypochlorite (1 mL/20 mg biomass) for 3 h at 37 °C, 170 rpm in a shaker incubator. The volume of the solution was made up to 25 mL using sterile distilled water, and the lead was quantified using AAS (Kushwaha et al. 2017).

#### Estimation of lead in various cell fractions

The bacterial cultures in MSM collected at regular intervals were adjusted to an optical density of 1 using sterile distilled water. The cultures were centrifuged at 8000 × g for 8 min to get the cell pellets. The pellets were suspended in 10 mM Tris–HCl buffer (pH = 8), and the cells were disrupted by ultrasonication. The lysates were centrifuged at 10,000 × g for 10 min to get the cytoplasmic fraction as supernatant. The pellets obtained were suspended in a protein-extraction solution (5% sodium dodecyl sulphate, 10 mM Tris–HCl, pH = 8) and centrifuged at 10,000 × g for 10 min to obtain the protein fraction as supernatant. The pellets obtained were digested with 4% sodium hypochlorite for 3 h at 37 °C, 170 rpm in a shaker incubator to get the non-protein fraction. The amount of lead in cytoplasmic, protein and non-protein fractions was analyzed using AAS (Mwandira et al. 2020).

### Statistical analysis

All the experiments were performed in identical triplicates (n = 3), and the graphs were plotted by taking the average of the three values and standard deviation as error bars. Unpaired sample T-test was performed using MS. Excel to find out significant (P < 0.05) increase in the PHA yield in test sample.

## Results and discussion

### Analysis of chemical constituents in the lake water sample

The water sample showed high COD, indicating many organic and inorganic oxidizable components. A low nitrate and nitrite concentration in the water sample shows that the lake water is low in inorganic nitrogen (Table 1). The majority of the PHA-producing bacteria are known to accumulate PHA during excess carbon sources and limiting nitrogen concentration in the media. Hence, the presence of high organic carbon and low nitrogen concentration limits the growth of non-PHA-producing bacteria. The nitrogen limitation induces stress in the bacterial cells and diverts organic carbon sources towards PHA production (Ahn et al. 2015). Hence, the lake water’s high carbon: nitrogen (C:N) ratio favours the growth of PHA-producing bacteria. A low BOD value of 4 mg/L was observed in lake water compared to the COD value. This indicates that most organic carbon in the water was not biologically oxidized and was not utilized for the growth of microorganisms. The limited nitrogen source reduced the growth of microorganisms, reducing the BOD of lake water (Table 1). The dissolved oxygen in the lake water was high above the permissible range (DO > 6 mg/L) for the polluted lake water (Vasistha and Ganguly 2020). Lake water had a low salinity of 0.03% and slightly higher total alkalinity compared to total acidity, indicating a high capacity of lake water to neutralize the influx of acidic components into the lake (Table 1).

**Table 1:** Concentration of chemical constituents in the lake water sample

Chemical constituents	values
COD	64 mg/L
BOD	4 mg/L
Dissolved oxygen	7.05 mg/L
Salinity	0.03%
Alkalinity	70 mg/L as CaCO_3_
Acidity	5 mg/L
Nitrates	0.2 mg/L
Nitrites	0.01 mg/L

### Growth and biosorption of lead in *Bacillus paramycoides*

**Fig. 1:**
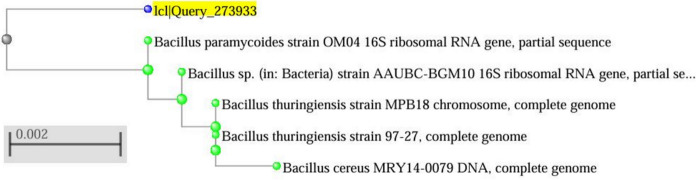
Distance tree of BLAST results showing the nearest neighbour of the bacterial isolate

**Fig. 2:**
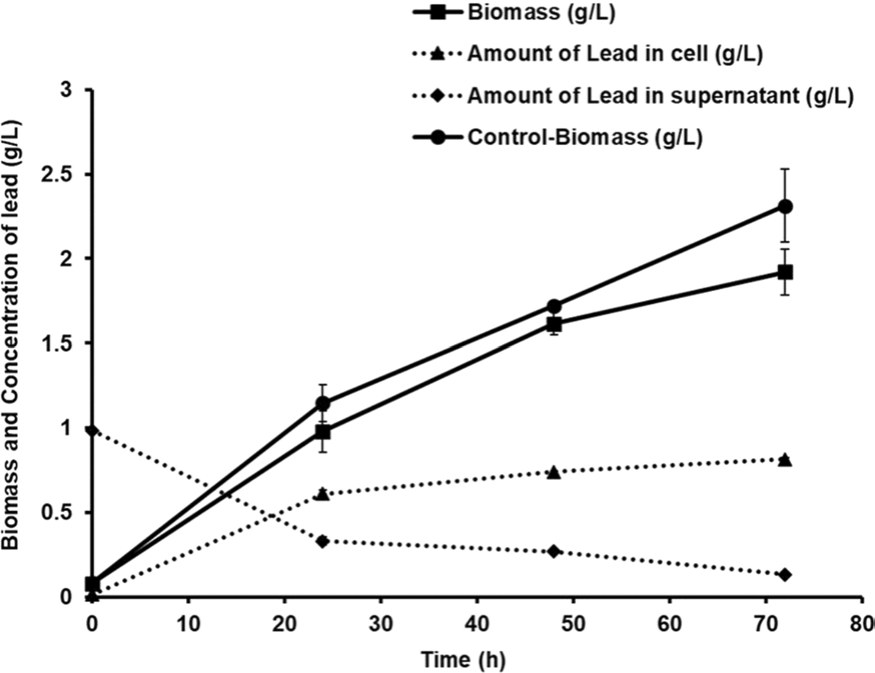
Growth and lead biosorption in *Bacillus paramycoides* cultured in MSM containing lead (1 g/L) at 30 °C, 170 rpm, and MSM without lead as a control

The BLAST analysis of the 16 s rRNA gene sequence showed a high sequence homology with *Bacillus paramycoides* strain OM04 with a Percentage identity, E-value, and query coverage of 99.51%, 0, and 100%, respectively. The total and the maximum score were 2612, indicating a high sequence similarity with the query sequence. The distance tree also indicates *Bacillus paramycoides* as the nearest neighbour (Fig. 1). Hence, the isolated bacterium is *Bacillus paramycoides*. The bacterial growth and lead biosorption were analyzed in *Bacillus paramycoides* cultured in MSM containing lead by taking cultures in MSM without lead as a control (Fig. 2). An increase in the bacterial biomass was observed in the presence of lead up to 0.98 g/L at 24 h of incubation from an initial biomass of 0.078 g/L during the time of inoculation. The bacterial biomass increased from 1.616 g/L at 48 h to 1.927 g/L at 72 h of incubation. The biomass in MSM containing lead was similar to the control at 24 h and 48 h of incubation, but a slight decrease in biomass was observed at 72 h in MSM containing lead compared to the control.

*Bacillus paramycoides* showed an initial lead concentration of 0.012 g/L inside the bacterial cells and a concentration of 0.986 g/L in the culture supernatant. The lead concentration inside the bacterial cells increased to 0.609 g/L at 24 h of incubation, a 50.7-fold increase from the initial lead concentration inside the bacterial cells. A high percentage biosorption of 62.1% cell dry weight (CDW) was achieved at 24 h of growth period. The lead in the supernatant saw a 2.9-fold reduction in the concentration of about 0.329 g/L compared to the initial lead concentration in the supernatant. A gradual increase in the lead concentration was observed inside the bacterial cells from 0.740 g/L at 48 h to 0.816 g/L at 72 h of incubation. The percentage biosorption was 45.7% CDW and 42.5% CDW at 48 h and 72 h, respectively. A prolonged incubation in MSM containing lead gradually reduced the lead concentration in the supernatant from 0.267 g/L at 48 h to 0.131 g/L at 72 h of incubation.

The lead biosorption was previously analyzed in *Bacillus cereus* and *Bacillus pumillus* dead biomass in batch adsorption and fixed-bed column studies. The lead biosorption in the *B. cereus* and *B. pumillus* reached 38.6 mg/g and 43 mg/g of bacterial biomass, respectively, at a pH of 6.0 and temperature of 25 °C in batch studies. The fixed-bed column studies using similar parameters at a flow rate of 0.5 mL/min led to biosorption of 59.2 mg/g in *B. cereus* and 66 mg/g in *B. pumillus* (Çolak et al. 2011). In the present study involving *Bacillus paramycoides*, a lead biosorption reached up to 423 mg/g in the biomass at 72 h of incubation, which was higher than the biosorption observed in living or dead biomass of *B. cereus*, *B. pumillus*, *B. xiamenensis* and *B. licheniformis* (Table 2). The increased lead biosorption in *B. paramycoides* is due to increased bacterial biomass at successive cultivation times (Fig. 2). The high nutrient availability during the early stages of cultivation enhances the growth of bacteria, which in turn provides a more absorptive surface for lead ions, causing an increase in lead biosorption. The technique can be upscaled in a bioreactor for industrial-scale lead removal from contaminated water without restricting bacterial growth and viability, as in bacterial immobilization and using dead biomass for lead biosorption. The lead biosorption using actively growing bacterial cultures avoids the problem of biosorbent saturation observed in immobilization techniques, and nutrient addition can improve efficiency.

**Table 2:** Comparison of *Bacillus paramycoides* lead ion biosorption with other bacteria

Bacterium	Conditions	Lead biosorption (mg/g)	Study
*Bacillus xiamenensis*	Live biomass	216.7	(Mohapatra et al. 2019)
*Bacillus xiamenensis*	Dead biomass	207.4	(Mohapatra et al. 2019)
*Bacillus licheniformis*	Immobilized using magnetic polyvinyl alcohol and sodium alginate mixture	113.8	(Wen et al. 2018)
*Bacillus cereus* SEM-15	Actively growing culture	80.28	(Li et al. 2023)
*Bacillus* strain MRS-2	Live biomass	99.35	(Hoyle-Gardner et al. 2021)
*Curtobacterium* sp. FM01	Dead biomass	186.6	(Masoumi et al. 2016)
*Bacillus paramycoides*	Actively growing culture	423	The present study

### Production of PHA in *Bacillus paramycoides*

**Fig. 3:**
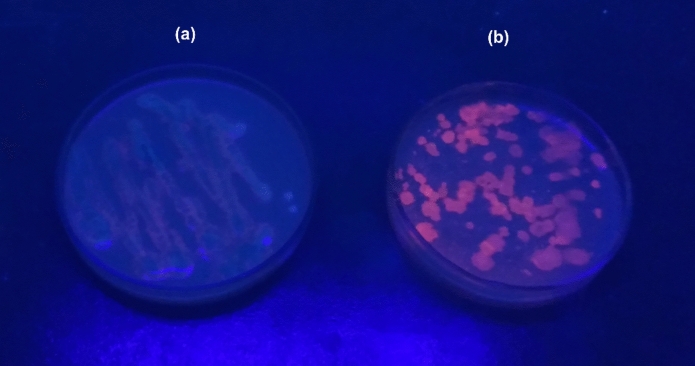
Nile red staining of the bacterial colonies. **a** *Escherichia coli* (Control) **(b)** *Bacillus paramycoides*

**Fig. 4:**
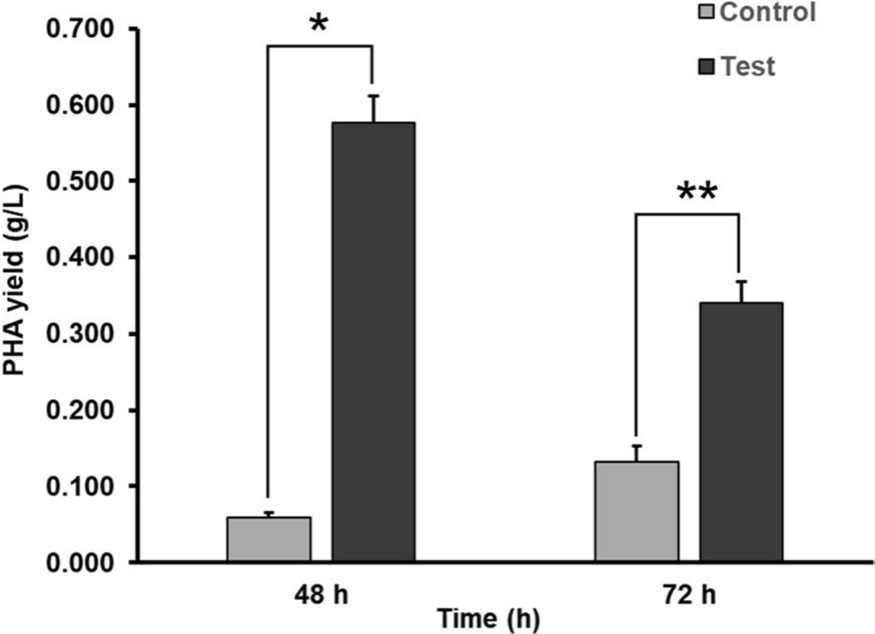
PHA production in *Bacillus paramycoides* cultured in MSM containing lead at 30 °C, 170 rpm at different incubation times by taking MSM without lead as a control. The asterisk (*) indicates a significant increase (*P = 0.00061, **P = 0.00035) in the PHA production

*Bacillus paramycoides* was detected for the PHA accumulation using the Nile red staining method by taking *Escherichia coli* as a control. An orange-coloured fluorescence was observed in the *Bacillus paramycoides* colonies in the media, showing the PHA production inside the bacteria (Fig. 3b). Whereas an absence of fluorescence observed in *Escherichia coli* indicates the absence of PHA inside the bacterial cells (Fig. 3a). The PHA production by *Bacillus paramycoides* was analyzed in MSM containing lead and MSM without lead as a control (Fig. 4). The volumetric PHA yield saw a 9.61-fold increase with a yield of 0.577 g/L compared to the control with a PHA yield of 0.06 g/L at 48 h of incubation. The PHA content in the bacterial cells in the presence of lead in the media reached up to 35.7% CDW compared to the control with a PHA content of 3.4% CDW. Further incubation to 72 h reduced the PHA yield at all bacterial growth conditions. Still, the PHA yield in the presence of lead was 2.57-fold high, with a yield of 0.34 g/L compared to the control with a PHA yield of 0.132 g/L. The PHA content in the bacterial cells was high at about 17.7% CDW in the presence of lead compared to a low PHA content of 5.7% CDW obtained in control. The unpaired sample T-test between the control and test sample showed a P-value of about 0.00061 and 0.00035 for 48 h and 72 h time points, respectively, which is less than 0.05. Hence, the presence of lead ions significantly enhanced the PHA production in bacteria. This is the first study showing biosorption of lead and simultaneous PHA production in *Bacillus paramycoides*. The study opens up possibilities for techno-economic analysis that may focus on cost reduction using inexpensive carbon sources for bacterial growth such as domestic waste and commercial effluents instead of sucrose and inexpensive nitrogen source such as urea instead of ammonium sulphate. Since lead removal from wastewater do not generate any revenue, integrating it with PHA production becomes profitable for the industries.

The lead ions (Pb^+2^) are known to elicit oxidative stress in bacterial cells by the generation of reactive oxygen species (ROS) such as superoxide (O_2_^−^), hydrogen peroxide (H_2_O_2_), hydroxyl radical (OH·) and singlet oxygen (^1^O_2_). ROS generation is mainly due to inhibiting cellular antioxidant enzymes such as superoxide dismutase, catalase, and peroxidase. These antioxidant enzymes rely on the reduced nicotinamide adenine dinucleotide phosphate (NADPH) as a cofactor for their redox activity (Sevak et al. 2021). The PHA is known to maintain redox balance in bacterial cells by storing excess NADPH and releasing it to boost antioxidant enzyme activity after polymer degradation into acetyl-CoA molecules (Obruca et al. 2018; García et al. 2018). In the present study involving *Bacillus paramycoides*, the oxidative stress induced by Pb^+2^ ions resulted in high accumulation of PHA in the bacterial cells at 48 h of incubation compared to the control. Therefore, lead did not inhibit *Bacillus paramycoides* growth at 48 h of incubation. However, a slight decrease in the biomass at 72 h of growth in the presence of lead is due to the PHA consumption in the bacterial cells during prolonged incubation for energy generation, causing reduced NADPH generation to maintain redox homeostasis and sustain growth during lead-induced oxidative stress (Figs. 2, 4).

### Bioadsorption of lead in various cell fractions of *Bacillus paramycoides*

**Fig. 5:**
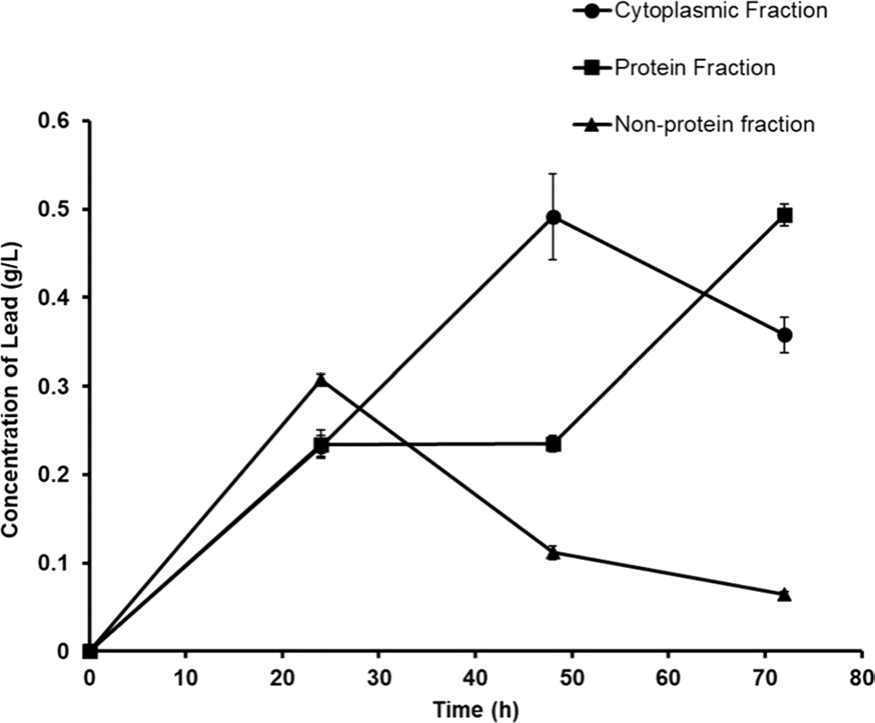
Lead bioadsorption in the different cellular fractions of *Bacillus paramycoides* cultured in MSM containing lead (1 g/L) at 30 °C, 170 rpm at various incubation time points

The bioadsorption of lead was analyzed in the cytoplasmic fraction containing cytoplasmic contents, genetic materials and soluble proteins, the protein fraction containing membrane-bound and insoluble cytoplasmic proteins, and the non-protein fraction containing bacterial cell wall materials and plasma membrane of *Bacillus paramycoides* cultured in MSM containing lead at various time points of incubation (Fig. 5). A slightly high lead concentration of about 0.307 g/L was observed in non-protein fraction compared to 0.232 g/L and 0.234 g/L in cytoplasmic and protein fractions at 24 h of incubation. The lead concentration drastically declined in the non-protein fraction during prolonged duration from 0.112 g/L at 48 h to 0.064 g/L at 72 h of incubation. The lead concentration in the cytoplasmic fraction increased to a maximum value of 0.492 g/L compared to the low lead concentration in protein and non-protein fractions. A reduction was observed in cytoplasmic lead concentration to 0.358 g/L at 72 h of incubation. Whereas, in the protein fraction, the lead concentration remained nearly constant at about 0.235 g/L till 48 h of incubation but significantly increased to a concentration of 0.493 g/L at a prolonged incubation of 72 h of growth.

The Pb^+2^ ions accumulate inside the cells through bioadsorption on the surface of various biological molecules such as proteins, lipids and other chemical components. The bioadsorption of Pb^+2^ ions is mainly due to the electrostatic attraction between ions and various cellular components. It occurs due to various exposed functional groups such as hydroxyl, carboxyl, sulfhydryl, sulphonate and phosphate with an electronegative atom(Ansari et al. 2011). A slight increase in lead bioadsorption was observed in the non-protein fraction containing plasma membrane and cell wall components compared to cytoplasmic and protein fractions at 24 h of incubation in *Bacillus paramycoides* (Fig. 5). It is due to the interaction of Pb^+2^ with the phosphate and carbonyl groups of membrane lipids and teichoic acid of the cell wall during passive diffusion of Pb^+2^ through the cell wall and plasma membrane(Ansari et al. 2011).

A high amount of lead was observed in the cytoplasmic fraction of bacteria at 48 h of incubation. It was followed by an increase in the PHA accumulation of up to 35.7% CDW in the bacterial cells. This is due to Pb^+2^ ions’ adsorption onto the PHA polymer (Fig. 4, Fig. 5). Lead ions’ adsorption onto the PHA polymer was earlier demonstrated in polyhydroxybutyrate (PHB) extracted from *Bacillus cereus* PW3A. This is due to the electrostatic attraction between the Pb^+2^ ion and oxygen atom of the carbonyl group, which is involved in the ester linkage between PHA monomers(Hungund et al. 2018). Hence, the adsorption of lead is directly proportional to the amount of PHA in the bacterial cells. The adsorption of lead to the PHA polymer also reduces the oxidative stress caused by free Pb^+2^ ions in the cytoplasm. Further incubation at 72 h reduced the lead concentration in the cytoplasmic fraction due to the PHA degradation. The PHA content in the cells decreased to 17.7% CDW, causing the intracellular lead to desorb from PHA polymer and get adsorbed to membrane-bound and insoluble cytoplasmic proteins, causing an increase in the lead concentration in protein fraction at 72 h of incubation (Figs. 4, 5).

### Characterization of the PHA using gas chromatography

**Fig. 6:**
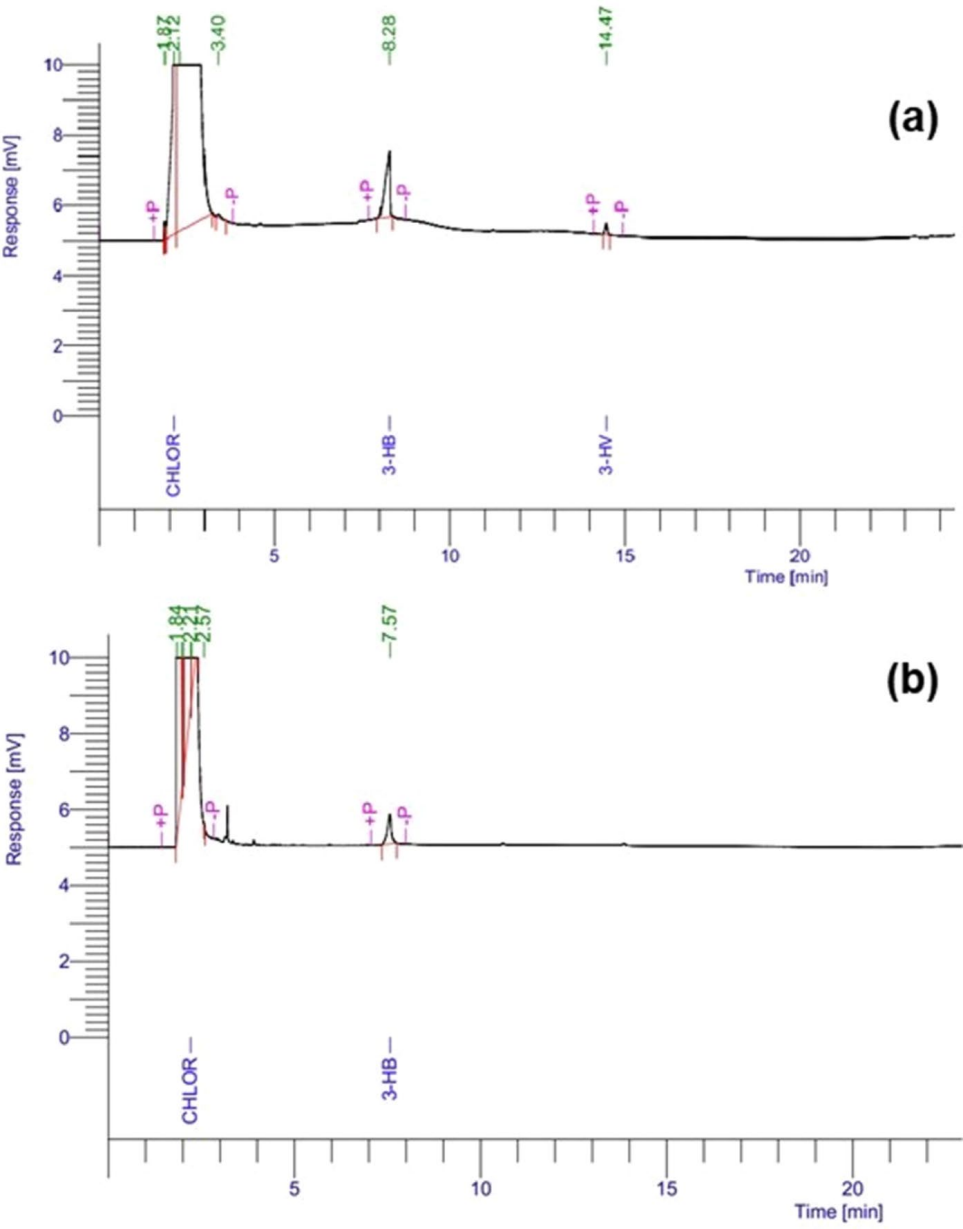
Gas chromatographic analysis of PHA **(a)** standard PHBV **(b)** PHA obtained from *Bacillus paramycoides*

The PHA polymer extracted from *Bacillus paramycoides* cultured in MSM for 72 h of incubation was analyzed for the monomeric composition by taking PHBV as a standard. The PHA sample obtained from the *Bacillus paramycoides* showed a peak at a retention time of 7.5 min, which is in the range of 3-hydroxybutyrate (3-HB) peak of the standard at a retention time of 8.2 min. A peak was absent in the PHA sample at a retention time of 14.4 min, corresponding to 3-hydroxyvalerate (3-HV) of the standard. Hence, the PHA polymer obtained from *Bacillus paramycoides* is a polyhydroxybutyrate (PHB) (Fig. 6).

## Conclusion

*Bacillus paramycoides* exhibited a high lead biosorption capability of about 423 mg/g of biomass at 72 h of incubation, which was not reported in previous studies. The lead ions induced oxidative stress in the bacteria, increasing the PHB production. This integrated process approach of lead removal and PHA production can be used in an industrial-scale without restricting bacterial growth and avoid biosorbent saturation.

## Supplementary Information

Below is the link to the electronic supplementary material.

Supplementary file1 (DOCX 23 KB)

## Data Availability

The datasets generated during the current study are available from the corresponding author upon reasonable request.
