# Supplementary material for: Dual-function strategy: simultaneous biopolymer production and lead biosorption by Bacillus paramycoides in single-batch fermentation process
Source: Biodegradation. 2025 Jul 22;36(4):65. doi: 10.1007/s10532-025-10160-2 (PMC12283803; doi:10.1007/s10532-025-10160-2)
Supplement: Supplementary file 1 — Supplementary file1 (DOCX 23 KB) [file 10532_2025_10160_MOESM1_ESM.docx]

**Supplementary Information**

**Dual-Function Strategy: Simultaneous Biopolymer Production and Lead Biosorption by *Bacillus paramycoides* in Single-Batch Fermentation Process**

Raghavendra Paduvari ^a^, Roopashri Arekal ^b^ and Divyashree Mysore Somashekara^a*^

^a^ *Department of Biotechnology, Manipal Institute of Technology, Manipal Academy of Higher Education, Manipal, 576104, Karnataka, India.*

^b^ *Department of Microbiology, Biotechnology and Food Technology, Bangalore University, Bengaluru, 560056, Karnataka, India.*

^*^Corresponding author: Email ID: [divyashree.ms@manipal.edu](mailto:divyashree.ms@manipal.edu)

1. **Materials and Methods**
   1. **Estimation of chemical oxygen demand (COD)**

The COD of the sample water was determined by Dichromate Reflux Method. About 25 mL of distilled water and water sample was taken in 2 separate conical flasks labelled as blank and test sample respectively. To each of conical flasks 500 mg of mercuric sulphate, 5 mL of sulphuric acid and silver sulphate solution was added with few glass beads. 25 mL of 0.25 N potassium dichromate was added and the flasks were attached to the condenser. About 30 mL of concentrated sulphuric acid was added into the flasks and refluxed for 2 h. The COD was estimated using a titrimetric method. The solution was titrated against 0.25 N ferrous ammonium sulphate solution after adding 1 mL ferroin indicator. The end-point was noted by color change from greenish blue to deep red(Greenberg et al. 1980).

- 1. **Estimation of biological oxygen demand (BOD)**

The dilution water was prepared by adding 2 mL of phosphate buffer, 22.5 % magnesium sulphate solution, 27.5 % calcium chloride, 0.15 % ferric chloride and seeding material (old domestic waste) in 2 L distilled water. The water sample was diluted to 50 % using dilution water in a 300 mL BOD bottle. The experiments were divided into two sets; the first set was used to estimate initial dissolved oxygen (DO) and other set was incubated for 5 days and then estimated for final DO. The DO in water sample was estimated using Winkler’s method and the difference between initial and final DO is used to estimate BOD of the water sample(Greenberg et al. 1980).

- 1. **Estimation of Dissolved oxygen**

The DO was estimated using the Winkler’s titration method. 2 mL of manganese sulphate solution and 2 mL of alkaline iodide-azide solution was added into the 300 mL water sample taken in a BOD bottle. The bottle was stoppered and manganese hydroxide precipitate was allowed to settle. 2 mL of concentrated sulphuric acid was added and shaken until precipitate was completely dissolved. 50 mL of the solution was pipetted out into conical flask and titrated against 0.01 N sodium thiosulphate solution until it becomes pale yellow, 1 mL of starch indicator was added and then titrated. The first disappearance of blue color indicates endpoint of reaction, the volume of titrant consumed was used to estimate the DO of water sample(Greenberg et al. 1980).

- 1. **Estimation of salinity**

Salinity was estimated using 4-Poles Graphite conductivity cell (HACH, model: CDC40101) as per the manufacturers’ instructions.

- 1. **Estimation of alkalinity**

To 20 mL of water sample, 2 drops of methyl orange indicator was added and titrated against 0.02 N hydrochloric acid. The endpoint was noted by color change from yellow to orange red. The average volume of HCl consumed from three trails was used to estimate alkalinity(Greenberg et al. 1980).

- 1. **Estimation of acidity**

The total acidity was estimated using titrimetric method. To 20 mL of sample water 5 drops of phenolphthalein indicator was added and titrated against 0.02 N sodium hydroxide solution. The appearance of a pale pink color denotes the endpoint of the reaction and the volume of the sodium hydroxide consumed was used in the estimation of acidity(Greenberg et al. 1980).

- 1. **Quantification of nitrates**

The nitrates were estimated by Devarda’s alloy method. 250 mL of water sample was taken a beaker and boiled to half its volume after adding 5 mL of sodium hydroxide solution to remove free ammonia. 1 g of Devarda’s alloy (50 % Copper, 45 % Aluminium, 5 % Zinc) was added and diluted to 200 mL with distilled water. It was then distilled in a distillation apparatus having a receiver containing 50 mL of 4 % Boric acid solution that absorbs ammonia. 25 mL of distillate was taken in 100 mL of standard flask, 2 mL Nessler’s reagent was added and made up to 100 mL using distilled water. The nitrates were estimated using colorimetric method at a wavelength of 420 nm(Greenberg et al. 1980).

- 1. **Quantification of nitrites**

The pH of the water sample was adjusted to 7 using 1 N hydrochloric acid and 1 N sodium hydroxide solution. 25 mL of the water sample was taken in a 100 mL standard flask and 1 mL of 0.3 % sulphanilic acid solution was added, mixed and kept for 10 min. 1 mL of 0.05 % 1-napthylamine hydrochloride and 1 mL of sodium acetate buffer was added and the volume was made up to 100 mL using distilled water. The concentration of nitrites was estimated using colorimetric method at a wavelength of 520 nm(Greenberg et al. 1980).

1. **References**

Greenberg AE, Connors JJ, Jenkins D (1980) Standard Methods For the Examination of Water and Wastewater, 15th edn. American Public Health Association, American Water Works Association, Water Pollution Control Federation, USA
